# Evaluation of the Ridaquick Rotavirus/Adenovirus Immuno-Chromatographic Assay in Real-Life Situation

**DOI:** 10.3390/pathogens10091213

**Published:** 2021-09-18

**Authors:** Francis Simo-Fouda, Laetitia Ninove, Léa Luciani, Christine Zandotti, Céline Gazin, Remi N. Charrel, Antoine Nougairède

**Affiliations:** 1 Unité des Virus Emergents (UVE: Aix Marseille Univ, IRD 190, INSERM 1207, IHU Méditerranée Infection), 13005 Marseille, France; francis-girauld.simo-fouda@etu.univ-amu.fr (F.S.-F.); laetitia.ninove@univ-amu.fr (L.N.); lea.luciani@ap-hm.fr (L.L.); christine.zandotti@ap-hm.fr (C.Z.); remi.charrel@univ-amu.fr (R.N.C.); 2 Institut Hospitalo-Universitaire (IHU) Méditerranée Infection, 13005 Marseille, France; celine.gazin@ap-hm.fr

**Keywords:** rotavirus, adenovirus, immunochrommatographic test, ICT, PCR

## Abstract

Immunochromatographic tests (ICT) are diagnostics tools providing rapid results without the need for specialized equipment. Our aim was to evaluate retrospectively the rotavirus and adenovirus ICT routinely used in the virology laboratory serving the University Hospital of Marseille, France. From January 2017 to March 2020, 715 stool specimens from patients were screened using the Ridaquick Rotavirus/Adenovirus Combi ICT (RR/AC ICT) and a commercially available multiplex PCR detection kit. Rotavirus was detected in 9.2% of specimens by PCR and 7.7% of specimens by RR/AC ICT while adenovirus was detected in 8.5% of specimens by PCR and 2.4% of specimens by RR/AC ICT. The RR/AC ICT parameters for rotavirus were 75.8% sensitivity, 99.2% specificity, 90.9% positive predictive value (PPV) and 97.6% negative predictive value (NPV). The RR/AC ICT parameters for adenovirus were 6.6% sensitivity, 98.0% specificity, 23.5% PPV and 91.8% NPV. While the ICT test may be suitable for rotavirus detection, a PCR–based assay is better adapted for adenovirus detection in stools.

## 1. Introduction

Diarrhea remains a frequent illness throughout the world and despite extensive use of diagnostic tools, the cause of many episodes often remains unresolved [1,2]. Studies on acute gastroenteritis recognized rotaviruses and noroviruses as the leading causes, followed by adenoviruses, sapoviruses and human astroviruses [3,4,5].

Several point-of-care immunochromatographic tests (ICT) for viral enteropathogens are commercially available [6]. ICT are attractive because each sample can be tested individually with results available in <30 min; moreover, they require neither specific equipment nor advanced personnel training [7,8]. However, ICT often suffer from low sensitivity, failing to produce a positive result when the viral loads of rotavirus and adenovirus are low [7]. In contrast, molecular detection of viral targets using PCR result in higher assay sensitivity and specificity, making them the ‘gold standard’ for the detection of viral enteric pathogens [9,10]. Few studies have evaluated the performances of the Ridaquick Rotavirus/Adenovirus Combi ICT (RR/AC ICT) [6,11,12]. The aim of the study was to retrospectively assess the sensitivity and specificity of the RR/AC ICT routinely used in point of care settings in comparison with the Fast Track Diagnostics (FTD) Viral Gastroenteritis assay (FTD-GA; Fast Track Diagnostics, Luxembourg), which is a commercial multiplex PCR assay.

## 2. Results

A total of 13,280 samples were received in the laboratory for detection of enteric viruses during the study period. Amongst these samples, 715 samples from 659 patients were tested using both assays and were included in this study. The median age was 2 years (range 1 day–98 years) with a female/male ratio of 1.0/1.1.

Of the 715 specimens, 7.7% (55/715) were positive for rotavirus using ICT and 9.2% (66/715) were positive using PCR (Table 1). Adenovirus was detected in 2.4% (17/715) of specimens using ICT and 8.5% (61/715) of specimens using PCR (Table 1). Using FTD-GA PCR assay as reference, there were more false positive than true positive detection for adenovirus.

Using FTD-GA PCR assay as reference, the performance of the ICT for detection of rotavirus and adenovirus in stool specimens are presented in Table 2. Overall, our results indicated acceptable performances for rotavirus detection; concordance between ICT and PCR was 97.1% (95% confidence interval: 95.8–98.3%). In contrast, the ICT performance for adenovirus detection was poor with low sensitivity (6.6%) and positive predictive value (PPV; 23.5%) although the result concordance between the assays was 90.2% (95% confidence interval: 88.0–92.4%). It is worth noticing that the FTD-GA PCR assay covers all genotypes of Human mastadenovirus A to G species [13].

Available cycle threshold values (Ct) of positive samples are shown in Table 3 (278/715 samples tested during corresponding period). For rotavirus, almost all samples tested positive (16/19) using the FTD-GA PCR assay were also positive with the ICT, with Ct values ranging from 15 to 25. In contrast, for adenovirus, only one sample with a Ct value of 12 was positive with both FTD-GA PCR assay and ICT. All the other PCR positive samples were ICT negative with Ct values ranging from 18 to 34.

## 3. Discussion

The FTD-GA PCR detected more rotavirus and adenovirus cases than the ICT although this was not unexpected. The ICT manufacturer reported rotavirus assay parameters at 97.8% sensitivity, 94.4% specificity, 93.8% PPV and 98.1% NPV [14]. In other studies performed in Ghana, Turkey and Italy that evaluated also the same RR/AC ICT, sensitivity for rotavirus ranged from 75% to 89% whereas specificity ranged from 72% to 100% [6,11,12]. Our results are in line with previous studies and confirm that this RR/AC ICT is valuable for rapid detection of rotaviruses [6,11,12].

For adenovirus, the manufacturer reported performance of 72.7% sensitivity, 98.2% specificity, 97.0% PPV and 80.9% NPV [14]. Sensitivity and PPV were much higher than those observed in our study (6.6% sensitivity and 23.5% PPV). In our study, the observed lack of sensitivity cannot be due to sampling and processing procedure since the samples were processed/stored within 4 h after collection. Moreover, our results are in the same order of magnitude with those previously reported with sensitivity ranging from 22.0% to 28.6% [6,12].

The highly discrepant performances claimed by the manufacturer raises the question of how was constituted its panel of clinical specimens.

The reason could reside in the failure to detect specific genotypes within human adenoviruses. Even though FTD-GA PCR covers all genotypes [13], the RR/AC ICT manufacturer did not provide details about possible restrictions in genotype detection. However, FTD-GA PCR may detect adenovirus genotypes that are bystanders and are not truly responsible for gastroenteritis. Interestingly, Banerjee A. et al. showed that another ICT test (VIKIA1 Rota-Adeno assay, Biomerieux, Craponne, France) that detects almost exclusively (98% of PCR–confirmed positive) enteric adenoviruses (genotype 40/41) missed 35.3% of adenovirus infection due to restricted target scope [15,16]. In addition, previous studies have showed a high frequency of non-enteric adenovirus in stools of patients admitted for acute gastroenteritis [17,18]. Other Rotavirus/Adenovirus ICT displayed sensitivities for enteric adenoviruses ranging from 71% to 85% [19,20]. However, when including non-enteric adenoviruses, their sensitivity dropped to a great extent [8,12,20,21]. The very low sensitivity observed is likely to result in the inability of ICT to detect adenovirus antigens in stool samples presenting with low viral loads as described [7]

In this context, several studies concluded that they are not suitable for the routine diagnosis of adenovirus in stools and first-line tests should be PCR assays. Our results support the same conclusions for the tested RR/AC ICT.

In conclusion, this study suggests that RR/AC ICT can be used as a point-of-care test for rotavirus detection, and that the observed result can be considered as definitive in the vast majority of cases, i.e., for patients without specific co-morbidities. In contrast, RR/AC ICT might not be well-suited as a first-line rapid screen for adenovirus in stools that can be successfully achieved by using a PCR–based assay for diagnosis.

## 4. Materials and Methods

### 4.1. Study Design

We performed a retrospective analysis of results from stool samples processed by trained laboratory professionals from the University Hospitals of Marseille, France. Samples were analysed from 1 January 2017 to 15 March 2020. Stool samples were collected and processed within 4 h after collection (24/7 activity for point-of-care laboratories). The inclusion criteria consisted of (i) any stool sample collected from an admitted or a hospitalized patient (ii) that was addressed to the point-of-care laboratory for detection of adenovirus or rotavirus, and (iii) that was subsequently transferred to the core laboratory for PCR detection or confirmation (see below). Performance of the Ridaquick Rotavirus/Adenovirus Combi ICT (R-Biopharm AG, Darmstadt, Germany) was compared to the Fast Track Diagnostics (FTD) Viral Gastroenteritis assay (FTD-GA; Fast Track Diagnostics, Luxembourg). Ct (cycle threshold) values provided by FTD-GA PCR could be retrieved from the laboratory files only for recent years (from 1 August 2018 to 15 March 2020 in our case).

### 4.2. Immunochromatographic Test

RR/AC ICT is a lateral-flow ICT, which uses labeled monoclonal antibodies against surface antigens of rotavirus and adenovirus. The standard operating procedure has been written according to the manufacturer’s instructions. One fresh aliquot of stools was tested using the RR/AC ICT.

### 4.3. Nucleic Acids Extraction

A 100 mg stool sample was diluted and mixed into 1ml sterile water; 190 µL of this suspension was transferred into a 2ml tube that contained 2 µL of FTD-GA PCR internal control, 10 µL of MS2/T4 bacteriophages (in-house internal controls [22]) and 200 µL of ATL buffer. This preparation was used for nucleic acid extraction with the automated extractor EZ1^®^ Advanced XL (Qiagen, Hilden, Germany) using EZ1 Virus Mini Kit v2.0 (Qiagen) following the manufacturer’s instructions with the elution volume set at 120 µL.

### 4.4. Rotavirus and Adenovirus PCR Detection

Rotavirus and adenovirus FTD PCRs were performed using FTD Viral Gastroenteritis assay (ref# FTD-3–32/64, Fast Track Diagnostics, Luxembourg). For a single reaction mix, PPMix volume was 0.75 µL; buffer 6.25 µL; enzyme 0.50 µL; samples 5 µL; plate were run on Roche LightCycler 480 real-time PCR (LC480). Cycling temperature was set to 15 min at 42 °C, 3 min at 94 °C, 40 cycles of 8 s at 94 °C, 34 s at 60 °C. Amplification curves were analyzed using Lightcycler^®^ 480 software version 1.5.1 with standardized procedure.

### 4.5. Statistics

SPSS statistical package release 17.0 (SPSS Inc., Chicago, IL, USA) was used for data/statistical analysis.

## Figures and Tables

**Table 1:** Comparative detection of rotavirus and adenovirus in stools.

Rotavirus			Adenovirus		
ICT	PCR	No. of Specimens	ICT	PCR	No. of Specimens
Positive	Positive	50	Positive	Positive	4
Positive	Negative	5	Positive	Negative	13
Negative	Positive	16	Negative	Positive	57
Negative	Negative	644	Negative	Negative	641
Total		715	Total		715

**Table 2:** Performances of RR/AC ICT.

	Sensitivity (%)	Specificity (%)	PPV (%)	NP (%)
Rotavirus ICT	75.8	99.2	90.9	97.6
Adenovirus ICT	6.6	98.0	23.5	91.8

FTD-GA PCR assay was used as reference method. PPV = Positive predictive value, NPV = Negative predictive value.

**Table 3:** Available data on positive samples (August 2018–March 2020).

Rotavirus						Adenovirus					
N.	Sex ^†^	Age (Years)	Sampling Month	PCR (Ct)	ICT	N.	Sex ^†^	Age (Years)	Sampling Month	PCR (Ct)	ICT
1	F	<1	January 2020	**Positive (15) ^‡^**	**Positive**	1	M	<1	February 2020	**Positive (12) ^‡^**	**Positive**
2	F	<1	March 2019	**Positive (17)**	**Positive**	2	M	<1	May 2019	**Positive (18)**	Negative
3	M	7	September 2018	**Positive (17)**	**Positive**	3	F	1	September 2019	**Positive (20)**	Negative
4	M	1	March 2020	**Positive (18)**	**Positive**	4	F	1	October 2019	**Positive (21)**	Negative
5	F	4	March 2019	**Positive (18)**	**Positive**	5	F	<1	February 2020	**Positive (23)**	Negative
6	M	<1	September 2018	**Positive (18)**	**Positive**	6	F	92	January 2020	**Positive (23)**	Negative
7	M	<1	April 2019	**Positive (19)**	**Positive**	7	F	<1	October 2018	**Positive (23)**	Negative
8	F	<1	October 2018	**Positive (19)**	**Positive**	8	M	<1	October 2019	**Positive (27)**	Negative
9	M	1	March 2019	**Positive (21)**	**Positive**	9	M	6	September 2019	**Positive (27)**	Negative
10	M	4	March 2020	**Positive (22)**	Negative	10	M	3	November 2019	**Positive (31)**	Negative
11	F	2	April 2019	**Positive (22)**	**Positive**	11	M	2	February 2020	**Positive (32)**	Negative
12	M	<1	March 2019	**Positive (22)**	**Positive**	12	M	1	March 2020	**Positive (32)**	Negative
13	F	1	March 2020	**Positive (23)**	**Positive**	13	M	2	January 2020	**Positive (33)**	Negative
14	M	<1	March 2019	**Positive (23)**	Negative	14	F	4	March 2019	**Positive (33)**	Negative
15	F	<1	March 2019	**Positive (23)**	**Positive**	15	M	1	October 2019	**Positive (33)**	Negative
16	M	<1	February 2019	**Positive (23)**	**Positive**	16	M	4	March 2020	**Positive (34)**	Negative
17	M	1	December 2019	**Positive (25)**	**Positive**	17	M	50	May 2019	Negative	**Positive**
18	M	1	May 2019	**Positive (25)**	**Positive**	18	M	<1	June 2019	Negative	**Positive**
19	F	2	March 2019	**Positive (26)**	Negative	19	F	23	August 2019	Negative	**Positive**
20	M	35	March 2019	Negative	**Positive**	20	M	1	October 2019	Negative	**Positive**
21	M	13	August 2019	Negative	**Positive**						
22	F	79	September 2019	Negative	**Positive**						
23	M	<1	October 2019	Negative	**Positive**						

^†^ F = Female, M = Male; ^‡^ In bold = positive results.

## Data Availability

All data are provided in the article.

## References

[1] Ayouni S., Estienney M., Hammami S., Neji Guediche M., Pothier P., Aouni M., Belliot G., de Rougemont A. (2016). Cosavirus, Salivirus and Bufavirus in Diarrheal Tunisian Infants. PLoS ONE.

[2] Smits S.L., Schapendonk C.M., van Beek J., Vennema H., Schurch A.C., Schipper D., Bodewes R., Haagmans B.L., Osterhaus A.D., Koopmans M.P. (2014). New viruses in idiopathic human diarrhea cases, the Netherlands. Emerg. Infect. Dis..

[3] Desselberger U. (2017). Viral gastroenteritis. Medicine.

[4] Sattar S.B.A., Singh S. (2020). Bacterial Gastroenteritis. StatPearls.

[5] Portal T.M., Reymao T.K.A., Quindere Neto G.A., Fiuza M., Teixeira D.M., Lima I.C.G., Sousa Junior E.C., Bandeira R.D.S., De Deus D.R., Justino M.C.A. (2019). Detection and genotyping of enteric viruses in hospitalized children with acute gastroenteritis in Belem, Brazil: Occurrence of adenovirus viremia by species F, types 40/41. J. Med. Virol..

[6] Weitzel T., Reither K., Mockenhaupt F.P., Stark K., Ignatius R., Saad E., Seidu-Korkor A., Bienzle U., Schreier E. (2007). Field evaluation of a rota- and adenovirus immunochromatographic assay using stool samples from children with acute diarrhea in Ghana. J. Clin. Microbiol..

[7] Jiang N., Shi L., Lin J., Zhang L., Peng Y., Sheng H., Wu P., Pan Q. (2018). Comparison of two different combined test strips with fluorescent microspheres or colored microspheres as tracers for rotavirus and adenovirus detection. Virol. J..

[8] Kim J., Kim H.S., Kim H.S., Kim J.S., Song W., Lee K.M., Lee S., Park K.U., Lee W., Hong Y.J. (2014). Evaluation of an immunochromatographic assay for the rapid and simultaneous detection of rotavirus and adenovirus in stool samples. Ann. Lab. Med..

[9] Bloomfield M.G., Balm M.N., Blackmore T.K. (2015). Molecular testing for viral and bacterial enteric pathogens: Gold standard for viruses, but don’t let culture go just yet?. Pathology.

[10] Moutelikova R., Dvorakova Heroldova M., Hola V., Sauer P., Prodelalova J. (2019). Human rotavirus A detection: Comparison of enzymatic immunoassay and rapid chromatographic test with two quantitative RT-PCR assays. Epidemiol. Mikrobiol. Imunol..

[11] Artiran S., Atalay A., Gokahmetoglu S., Ozturk M.A., Balci N., Cakir N., Kilic H., Durmaz R. (2017). Investigation of Rotavirus with Various Methods in Children with Acute Gastroenteritis and Determination of Its Molecular Epidemiology in Kayseri Province, Turkey. J. Clin. Lab. Anal..

[12] Rovida F., Campanini G., Sarasini A., Adzasehoun K.M., Piralla A., Baldanti F. (2013). Comparison of immunologic and molecular assays for the diagnosis of gastrointestinal viral infections. Diagn. Microbiol. Infect. Dis..

[13] Sciandra I., Piccioni L., Coltella L., Ranno S., Giannelli G., Falasca F., Antonelli G., Concato C., Turriziani O. (2020). Comparative analysis of 2 commercial molecular tests for the detection of gastroenteric viruses on stool samples. Diagn. Microbiol. Infect. Dis..

[14] R-Biopharm AG RIDA^®^QUICK Rotavirus/Adenovirus Combi (Cassettes). https://clinical.r-biopharm.com/products/ridaquick-rotavirusadenovirus-combi-cassettes/.

[15] Banerjee A., De P., Manna B., Chawla-Sarkar M. (2017). Molecular characterization of enteric adenovirus genotypes 40 and 41 identified in children with acute gastroenteritis in Kolkata, India during 2013–2014. J. Med. Virol..

[16] Verma H., Chitambar S.D., Varanasi G. (2009). Identification and characterization of enteric adenoviruses in infants and children hospitalized for acute gastroenteritis. J. Med. Virol..

[17] Wu B.S., Huang Z.M., Weng Y.W., Chen F.Q., Zhang Y.L., Lin W.D., Yu T.T. (2019). Prevalence and Genotypes of Rotavirus A and Human Adenovirus among Hospitalized Children with Acute Gastroenteritis in Fujian, China, 2009–2017. Biomed. Environ. Sci..

[18] Gelaw A., Pietsch C., Liebert U.G. (2019). Genetic diversity of human adenovirus and human astrovirus in children with acute gastroenteritis in Northwest Ethiopia. Arch. Virol..

[19] Gonzalez-Serrano L., Munoz-Algarra M., Gonzalez-Sanz R., Portero-Azorin M.F., Amaro M.J., Higueras P., Cabrerizo M. (2020). Viral gastroenteritis in hospitalized patients: Evaluation of immunochromatographic methods for rapid detection in stool samples. J. Clin. Virol..

[20] Kaplon J., Thery L., Bidalot M., Grangier N., Frappier J., Aho Glele L.S., de Rougemont A., Ambert-Balay K. (2020). Diagnostic Accuracy of Four Commercial Triplex Immunochromatographic Tests for Rapid Detection of Rotavirus, Adenovirus, and Norovirus in Human Stool Samples. J. Clin. Microbiol..

[21] Kas M.P., Maure T., Soli K.W., Umezaki M., Morita A., Bebes S., Jonduo M.H., Larkins J.A., Luang-Suarkia D., Siba P.M. (2013). Evaluation of a rapid immunochromatographic assay for the detection of rotavirus, norovirus and adenovirus from children hospitalized with acute watery diarrhea. Papua N. Guin. Med. J..

[22] Ninove L., Nougairede A., Gazin C., Thirion L., Delogu I., Zandotti C., Charrel R.N., De Lamballerie X. (2011). RNA and DNA bacteriophages as molecular diagnosis controls in clinical virology: A comprehensive study of more than 45,000 routine PCR tests. PLoS ONE.

